# Effect of hot-pressing temperature on the structure and physico-mechanical properties of kapok fiber nonwoven mats

**DOI:** 10.1039/d6ra07101e

**Published:** 2026-10-21

**Authors:** M. A. M. Mohd Idrus, Md. Saiful Islam, Mohd Yuzri Mohd Yusop, Zulzamri Salleh

**Affiliations:** a Universiti Kuala Lumpur, Malaysian Institute of Marine Engineering Technology (UniKL MIMET), Dataran Industri Teknologi Kejuruteraan Marin Bandar Teknologi Maritim Jalan Pantai Remis Lumut 32200 Perak Malaysia msaifuli2007@gmail.com; b Bangladesh Army University of Engineering & Technology Bangladesh

## Abstract

Kapok fiber (KF) has gained increasing attention due to its lightweight, hollow structure and lignocellulosic nature, with excellent buoyancy, thermal insulation, and sustainability characteristics. However, it presents significant challenges during the carding process and nonwoven mat formation owing to its short fiber length and poor cohesion. Limited studies have systematically investigated the effect of controlled hot-pressing temperature on binder-free KF nonwoven mats. Therefore, this study examines the influence of hot-pressing temperature (140, 150, 160, and 170 °C) on their physical and mechanical properties. The KF nonwoven mats were fabricated using an Ashford drum carder followed by hydraulic hot pressing. Increasing the hot-pressing temperature from 140 to 170 °C reduced Grammage (GSM) and thickness from 30.08 to 28.16 g m^−2^ and 0.326 to 0.250 mm, respectively, while increasing density, tensile strength, and tensile modulus to 0.450 g cm^−3^, 50.76 MPa, and 6622.7 MPa, respectively, due to enhanced fiber consolidation. Among the tested conditions, 170 °C provided the highest mechanical performance and most effective fiber consolidation. Surface morphology was analyzed using a tabletop microscope and the results revealed a progressively more compact and smoother fiber structure at higher hot-pressing temperatures. Fourier transform infrared (FTIR) spectroscopy indicated a change in the intensity of hydroxyl (–OH) absorption bands with increasing temperature, suggesting partial thermal modification of the fiber surface. Moreover, tensile strength and tensile modulus increased with increasing hot-pressing temperature. A one-way analysis of variance (ANOVA) confirmed that hot-pressing temperature had a statistically significant effect on the physical and mechanical properties of the nonwoven mats. This work provides valuable insights into the sustainable utilization of KF and offers practical guidance for the industrial production of high-performance, eco-friendly nonwoven materials.

## Abstract 2

Kapok fibres (KF) were carded and hot-pressed at 170 °C for 10 min to produce a nonwoven mat with a tensile strength of 50.76 MPa, demonstrating its potential as a core material for fibreglass boat structures.

## Graphical abstract

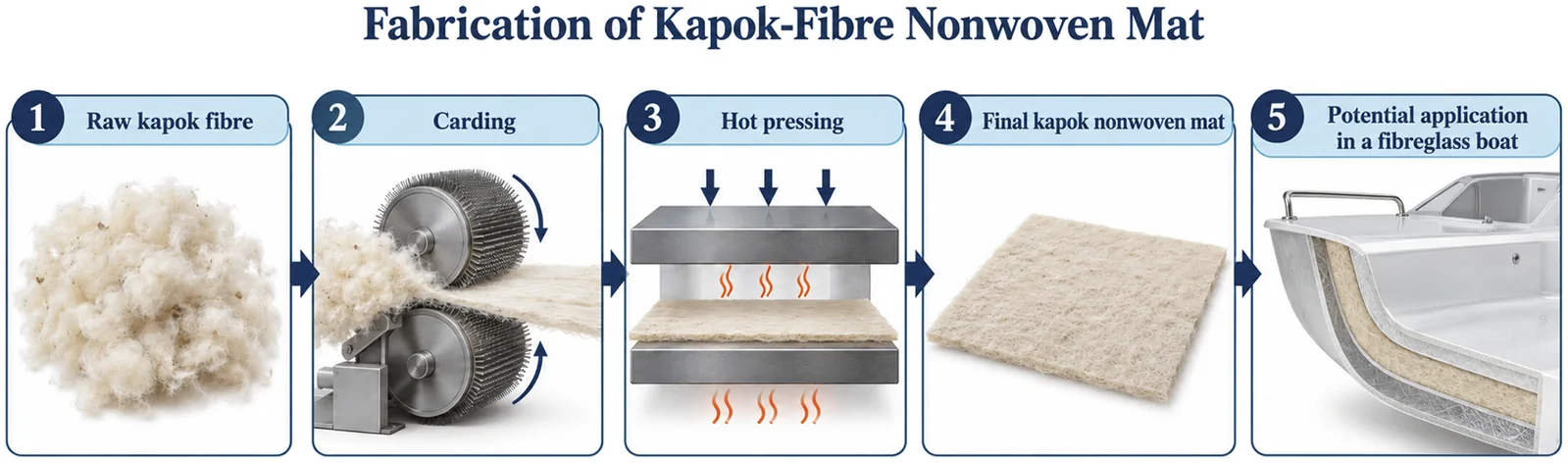

## 1. Introduction

The KF is a lignocellulosic natural fiber obtained from the seed hairs of *Ceiba pentandra*. It is distinguished by low density, high hollowness, and hydrophobic–oleophilic characteristics. KF is a lightweight, biodegradable, and water-resistant natural material that is widely used in sustainable applications. These unique properties make it highly suitable for applications such as oil sorption, thermal insulation, buoyancy materials, and sustainable nonwoven products.^1,2^ Due to its exceptional softness, buoyancy, and hypoallergenic nature, it is extensively applied in bedding products such as pillows and mattresses, as well as in thermal insulation, life jackets, furniture cushioning, and eco-friendly textile blends.^2^ Its renewable and chemical-free production process makes it a strong alternative to synthetic fibers, supporting reduced environmental impact and growing demand for sustainable materials in global markets.

In response to growing environmental concerns and the demand for biodegradable materials, KF has attracted increasing attention as a potential alternative to synthetic fibers in nonwoven manufacturing.^3^ However, despite its advantages, the industrial utilization of KF remains limited due to its smooth surface, short staple length, low inter-fiber cohesion, and high flyability, which create significant challenges during mechanical processing, particularly during web formation.^4^ The carding process is a critical stage in nonwoven production, where fibers are opened, individualized, and aligned to form a coherent web. The quality of the carded web directly affects final product performance, including uniformity, strength, and structural integrity.^5^ While carding parameters for conventional fibers such as cotton and polyester have been extensively studied and optimized, these conditions are not directly applicable to kapok fiber due to its distinct physical and morphological characteristics. Inappropriate carding conditions often result in excessive fiber loss, nep formation, web irregularity, and reduced consolidation capacity, thereby limiting its large-scale applicability.^6^ Therefore, optimization of the carding process is essential to improve fiber control, minimize material loss, and achieve uniform web formation. Key parameters such as feed rate, cylinder speed, doffer speed, and carding intensity must be carefully adjusted to balance fiber opening and web cohesion. A systematic investigation of these parameters is necessary to establish an optimal processing window tailored specifically to KF.

The carding process is identified as a primary method for converting raw KF into uniform nonwoven webs due to its superior ability to enhance fiber alignment, distribution, and web consistency compared to other mechanical techniques. Carding effectively opens and individualizes fibers while preserving the hollow and delicate morphology of kapok, which is essential for maintaining its low density and excellent thermal insulation properties. In contrast, preliminary trials with needle punching resulted in fiber scattering, poor entanglement, and significant material loss, indicating its unsuitability for low-cohesion kapok fibers.^7^ An Ashford Drum Carder was therefore used, offering controlled and gentle processing that minimizes fiber damage while enabling scalable web formation. Although minor surface alteration may occur, the resulting carded webs show improved uniformity and are compatible with subsequent thermal consolidation or hot-pressing processes without chemical binders.^8^ Furthermore, carding improves fiber orientation and surface exposure, enhancing adhesion with reinforcement materials such as fiberglass in hybrid composites. Previous studies shown that carded kapok webs processed under controlled thermal conditions (around 170 °C and 1000 psi) achieve optimal tensile strength and uniform thickness, making them suitable for lightweight marine composite applications.^9,10^ Overall, the carding process provides an effective balance between fiber preservation, structural integrity, and sustainability, enabling the development of biodegradable kapok-based hybrid composites for insulation and marine structural applications, while retaining key thermal and acoustic insulation benefits reported in kapok-based nonwoven materials.^11^

The scope of this study is to investigate the influence of hot-pressing temperature on the properties of KF nonwoven mat formation. The study also includes statistical analysis to confirm that hot-pressing temperature has a statistically significant effect on the physical and mechanical properties of the nonwoven mats. Polyethylene terephthalate (PET) is a widely used thermoplastic polymer known for its excellent mechanical strength, chemical resistance, dimensional stability, and recyclability. PET films are commonly used as lightweight, durable, and cost-effective release layers during hot pressing because they facilitate smooth separation from press plates and help maintain uniform heat and pressure distribution. Additionally, their smooth and inert surface facilitates easy removal of the consolidated mat while preserving its morphology.^12,13^ In the present study, PET films were used only as temporary, reusable layers between the nonwoven web and press plates; they were removed after pressing and were not incorporated into the specimens as a matrix or binder.

Although KF has attracted considerable interest as a sustainable natural fiber, studies on the fabrication of binder-free KF nonwoven mats and the influence of hot-pressing temperature on their structural and mechanical performance remain limited. The novelty of this work lies in the successful fabrication of KF nonwoven mats using an Ashford drum carder followed by hydraulic hot pressing, together with a systematic investigation of the effect of hot-pressing temperature on their physical, morphological, and mechanical properties. Therefore, the aim of this study is to evaluate the influence of hot-pressing temperature on the performance of KF nonwoven mats through measurements of GSM, thickness, density, surface morphology, and tensile properties, supported by statistical analysis (ANOVA).

## 2. Materials and methods

### 2.1 Materials

KFs were used as the natural fiber material due to their low density, high porosity and hollow tubular structure. A commercial biaxially oriented polyethylene terephthalate (PET)^14–16^ film with an oriented semi-crystalline structure, supplied by Penfiber Sdn. Berhad (Toray), Prai, Penang, Malaysia, was used solely as a reusable release/separation layer during hot pressing. The film was supplied in roll form, cut to size, and placed above and below the carded kapok web. The nominal film thickness was 38 µm (0.038 mm). The PET films prevented direct adhesion of the material to the upper and lower heated press plates and facilitated removal of the consolidated sheet. It was not function as a matrix or binder.

### 2.2. Kapok web production

KFs were processed using an Ashford drum carder to produce nonwoven webs. A small Ashford wide drum carder with a working width of 30 cm was employed for web formation. Raw fibers were opened and cleaned, and 100% kapok fiber was used without blending with other fibers. Batches weighing 40 g were carded and the resulting webs were collected and dried in an oven at 60 °C for 24 h. Each carded web measured 250 mm × 250 mm and had a gross as-carded mass of approximately 8 g, corresponding to an initial areal mass of approximately 128 g m^−2^. This initial gross mass is distinct from the final hot-pressed specimen mass used to calculate Table 3. For a final specimen area of 0.0625 m^2^, the reported GSM values correspond to final masses of approximately 1.88, 1.86, 1.84 and 1.76 g at 140, 150, 160 and 170 °C, respectively. Fig. 1 shows the sequence from raw kapok fiber to carded web; the close-up panel is labelled (e).

**Fig. 1:**
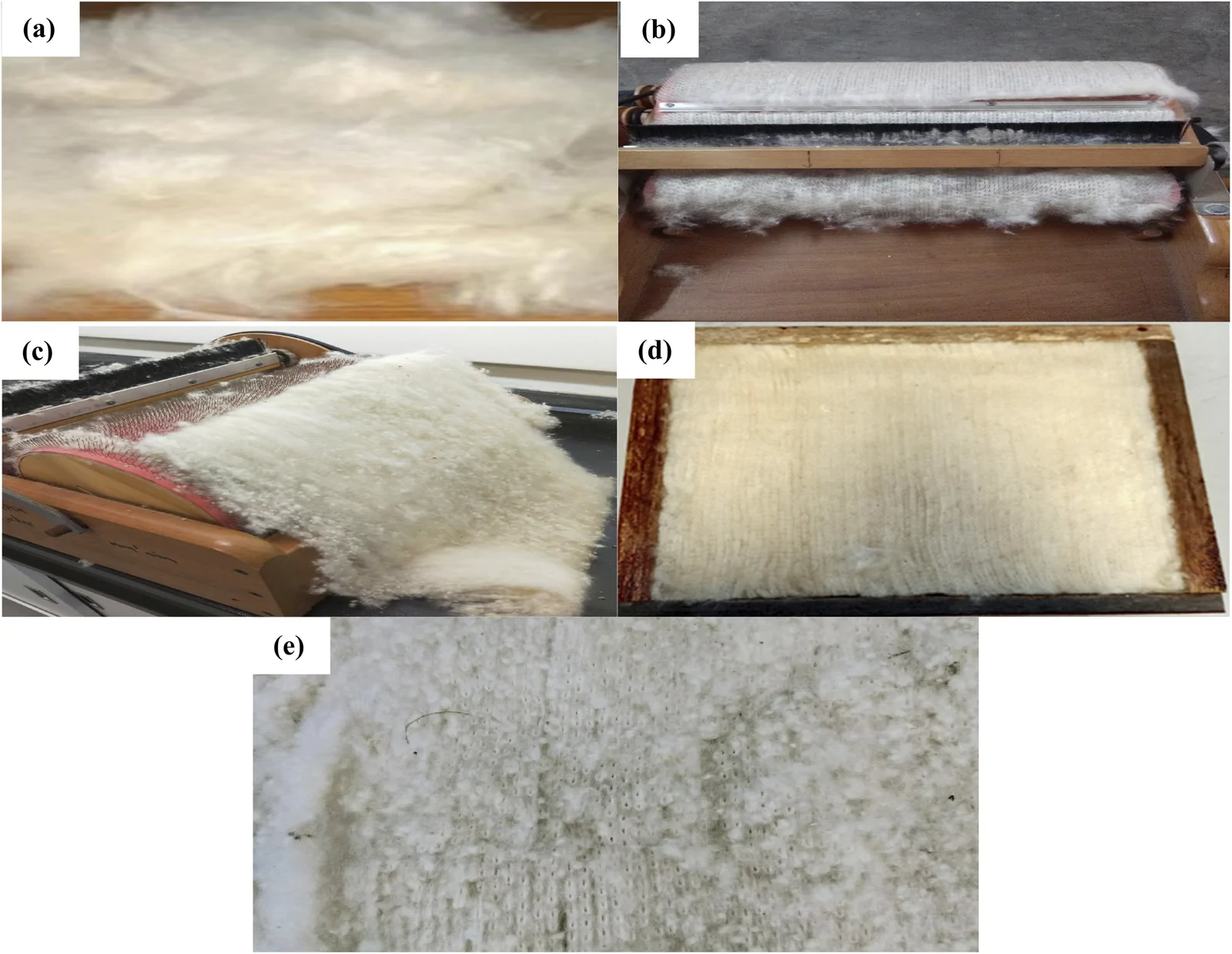
(a) Raw KF; (b) KF during carding; (c) KF web produced using an Ashford drum carder; (d) carded KF web showing the aligned web structure; and (e) a close-up view of the carded web surface showing a randomly oriented fibrous network.

### 2.3. Kapok nonwoven mat production

KF webs were consolidated using a hydraulic hot press at 140, 150, 160, and 170 °C under a pressure of 1000 psi (6.894 MPa). This process densifies the loosely arranged KF into a cohesive mat with improved mechanical performance, making it suitable for composite applications.^17^ For each run, the operator waited until the press reached the set temperature, after which the 10-min dwell period was started. After the dwell stage, the assembly was allowed to cool for 15 min. The heating rate and numerical cooling rate was not recorded. After each hot-pressing cycle and subsequent cooling, the PET films were separated from both surfaces of the consolidated specimen. The films were retained and reused in subsequent hot-pressing cycles rather than discarded. No PET layer remained attached to or embedded within the specimens used for mechanical characterization. Consequently, PET was not a load-bearing polymer matrix and did not form part of the final material whose mechanical properties were evaluated. The term “binder-free” refers to the absence of any intentionally incorporated polymeric binder within the consolidated kapok-fiber structure.

As shown in Fig. 2, a uniform KF web obtained after carding (Fig. 2a) was combined with PET films cut from a roll (Fig. 2b) and subjected to hydraulic hot-pressing (Fig. 2c). PET films were positioned on both sides of the fiber web during pressing to protect the material and promote the formation of a uniform nonwoven mat (Fig. 2d).

**Fig. 2:**
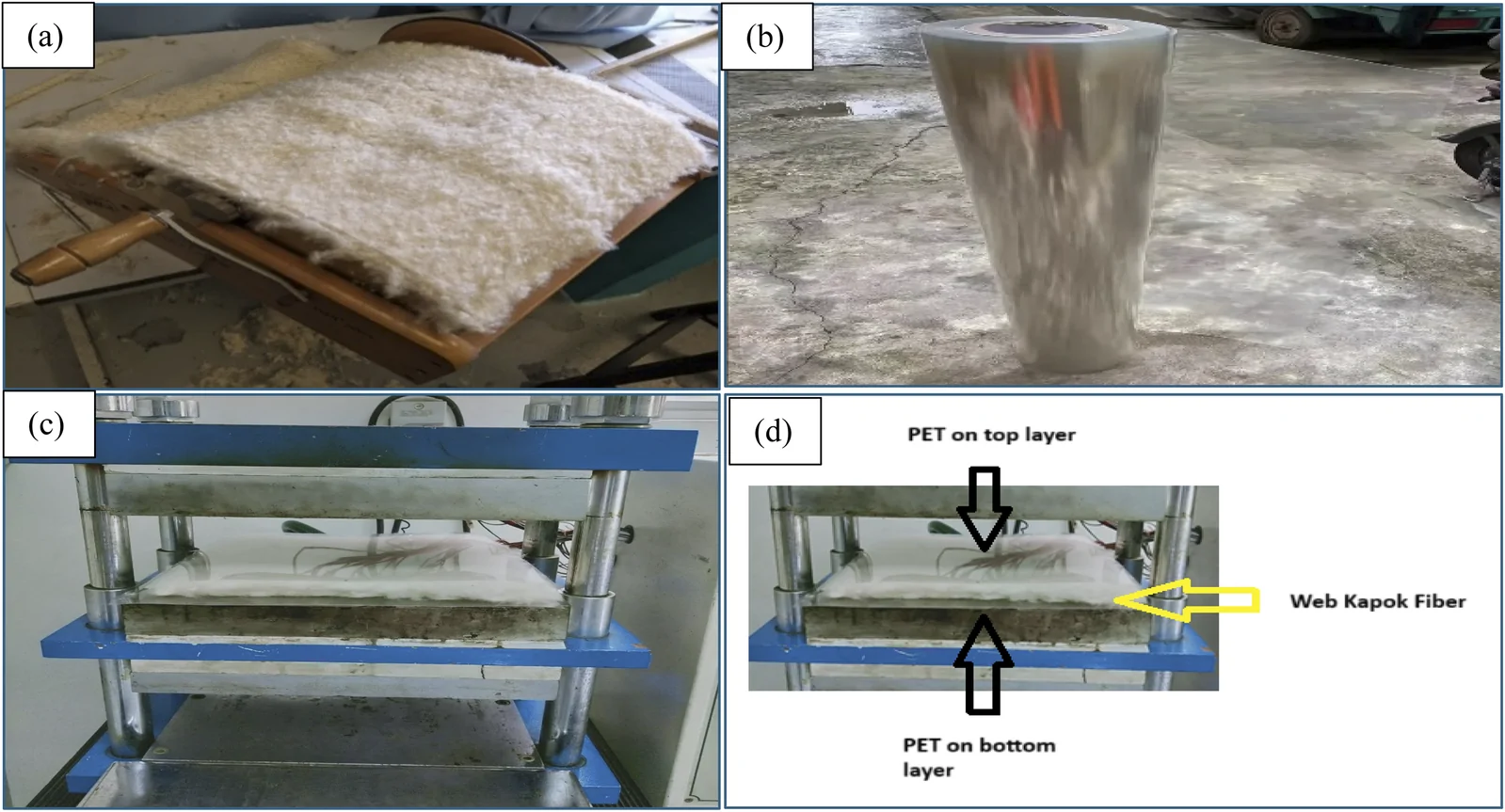
(a) Uniform KF web after carding; (b) PET film cut from a roll for use as a temporary reusable release layer; (c) hydraulic hot pressing of the carded web; and (d) PET films positioned as protective top and bottom separation layers during pressing.

Sample preparation and identification in this study are presented in Table 1.

**Table 1:** Sample designation and hot-pressing temperature of KF nonwoven mats

Sample code	Hot-pressing temperature (°C)
T140	140
T150	150
T160	160
T170	170

### 2.4. Characterization

The characterization of the KF nonwoven mat was carried out using several analytical techniques to evaluate its physical, chemical, morphological, and mechanical properties. FTIR analysis was performed using an FTIR spectrometer (Model 100 series, PerkinElmer) within the scanning range of 4000–400 cm^−1^ to identify the functional groups and chemical composition of the fiber before and after carding. The GSM of the fiber mat was determined according to ASTM D3776 using a precision electronic balance and expressed in g m^−2^. Thickness was measured using a Mitutoyo digital thickness gauge according to ASTM D1777; the applied gauge pressure and foot area were 4.14 ± 0.21 kPa (0.60 ± 0.03 psi) and 28.7 ± 0.02 mm. Mechanical properties, including tensile strength and tensile modulus, were evaluated according to ASTM D5035 using a Universal Testing Machine (UTM) (Model: Instron 3365, USA) under controlled testing conditions. Surface structure of the fibers was examined using a tabletop microscope (Model: Hitachi TM4000Plus, Japan) to observe fiber distribution, orientation, and web uniformity. Furthermore, statistical analysis was performed using ten replicates for each temperature condition. One-way ANOVA was performed at *α* = 0.05, followed by Tukey's HSD *post–hoc* test for pairwise comparisons.

Ten specimens were tested for each temperature. One-way ANOVA was conducted at *α* = 0.05, followed by Tukey's HSD *post–hoc* comparisons at *α* = 0.05.

## 3. Result and discussions

### 3.1. Physical and surface properties

The morphological changes in KFs made by hot pressing were examined using a tabletop microscope to evaluate KFs surface morphology, compaction, and structural integrity.

A preliminary trial at 70 °C was conducted for process selection, where the fibers remained relatively loose with noticeable voids between them. Therefore, only 140, 150, 160, and 170 °C were included in the formal statistical analysis. To achieve effective compaction and densification of KF for composite applications, processing temperatures significantly higher than 70 °C are required. This observation is novel, as previous research has not reported the effect of low-temperature hot-pressing on kapok fibers. For instance, Zhang *et al.* (2022) studied waste hemp fiber/PLA composites hot-pressed at 140 °C, focusing mainly on sound absorption rather than the structural and mechanical effects of low-temperature pressing.^18^ Similarly, Wang *et al.* (2023) produced kapok/waste silk non-woven materials *via* hot-pressing yet did not explore the specific effects of pressing at low temperatures (*e.g.*, 140–150 °C) on fiber morphology or mechanical properties.^19^ Based on these findings, further experiments were performed at 140 °C, 150 °C, 160 °C, 170 °C, and 180 °C. However, exposure to 180 °C caused significant thermal degradation and fiber charring, indicating excessive heat-induced damage. Therefore, samples processed at this temperature were excluded from subsequent analyses.

Fig. 3a represents the initial state of the KF web, displaying a loose and fluffy morphology with substantial inter-fiber gaps. The high porosity of raw KF makes them unsuitable for composite applications without further treatment. Conversely, Fig. 3b demonstrates the effects of hot-pressing, where the application of heat and pressure above 70 °C led to densification, improved fiber connecting and reduced porosity. The fibers became more compact, forming a nonwoven structure with better mechanical integrity. The microstructural changes in nonwoven KF subjected to hot-pressing at different temperatures, ranging from 140 °C to 170 °C as shown in Fig. 3c–f showed. The micrographs provide insights into fiber morphology, thermal effects, and densification behavior at each processing condition. At 140 °C, the fibers exhibit a relatively uniform and smooth surface, indicating the initial stage of fiber interlocking and structural consolidation. The observed colour change suggests the onset of thermal softening and densification, as previously reported in natural fiber processing studies.^20,21^ As the temperature increases to 150 °C, fiber densification is more pronounced, with enhanced fiber-to-fiber interactions and reduced voids, signifying improved mechanical interlocking. At 160 °C, the fibers display darker coloration and an increasingly cohesive structure, indicating intensified thermal consolidation. This transformation is attributed to localized fiber fusion and polymerization effects, consistent with findings from previous research on lignocellulosic fiber composites.^22^ The combination of high temperature and pressure promotes fiber compaction, reducing porosity and increasing the rigidity of the nonwoven matrix. However, at 170 °C, the fibers begin to exhibit visible thermal degradation, as evidenced by darkened regions and surface irregularities. The excessive heat initiates fiber burning and decomposition, similar to observations in other natural fiber-based materials subjected to extreme thermal processing. The finding also highlights 170 °C as the upper processing limit for maintaining fiber integrity while achieving optimal mechanical properties. The findings from this stage of this study provide important data for optimizing kapok fiber-based composites in industrial applications. The 140 °C–170 °C range is identified as the ideal processing window for producing high-performance web kapok, bio-based composites. Thus, this material offers sustainable alternatives for marine applications, automotive components, and eco-friendly packaging.^23,24^

**Fig. 3:**
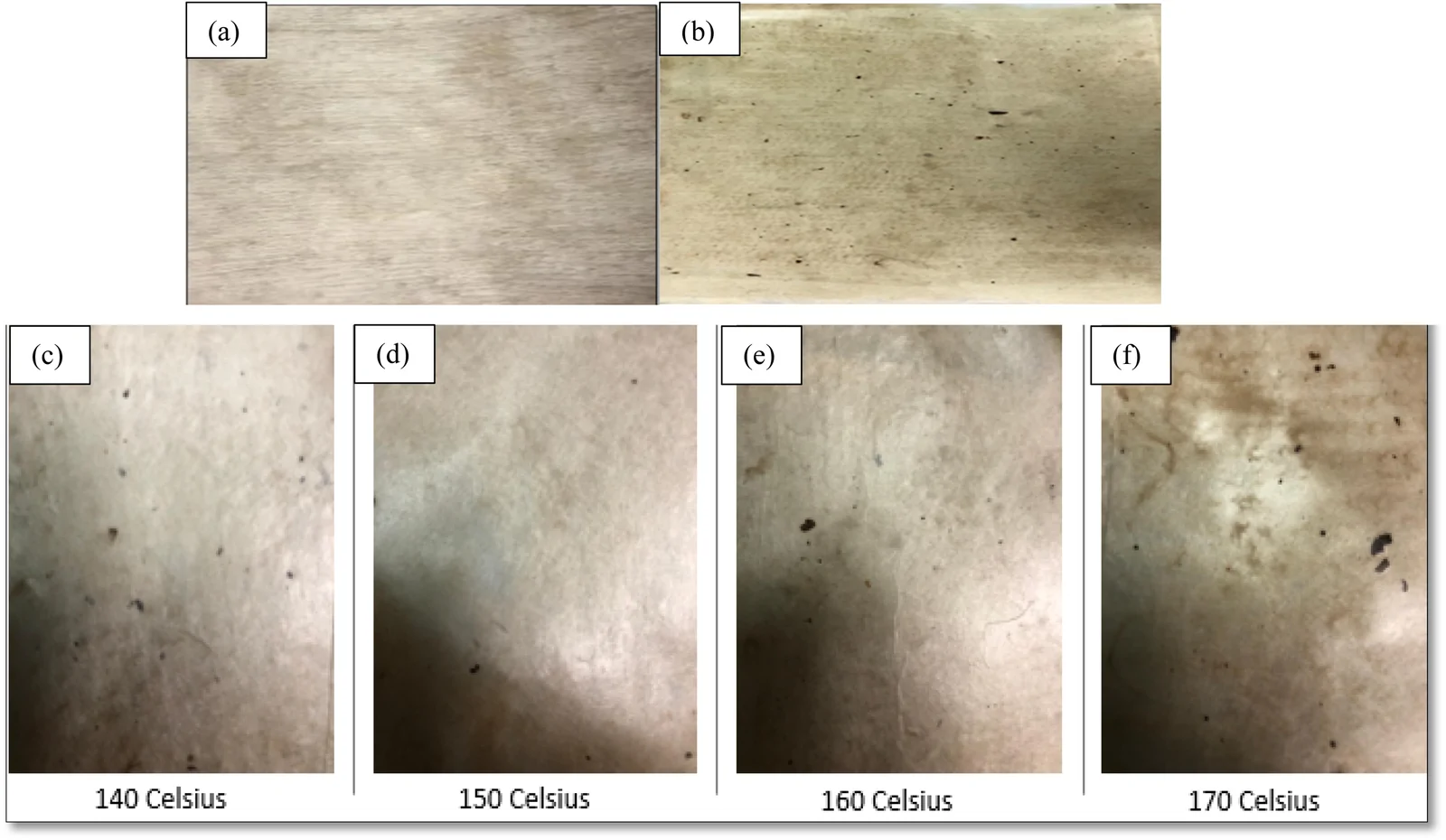
Microstructural images of KF web before and after hot pressing: (a) KF web before hot pressing, (b) KF nonwoven mat after hot pressing, and (c–f) KF nonwoven mats hot-pressed at (c) 140 °C, (d) 150 °C, (e) 160 °C, and (f) 170 °C.

### 3.2. FT-IR analysis

FTIR was used to investigate the chemical structure of raw KF and mats, as illustrated in Fig. 4. The FTIR spectra of raw and hot-pressed nonwoven KF samples (T140, T150, T160, and T170) show a broad absorption band between 3478 and 3330 cm^−1^, which is indicative of hydroxyl groups (–OH) present in cellulose, a major component of KFs. In the heat-treated samples, this band shifts to 3361, 3330, and 3434 cm^−1^, indicating a significant reduction in hydroxyl groups due to heat treatment. A very clear absorption peak observed around 1732–1728 cm^−1^ is attributed to the C=O stretching vibration of acetyl groups in hemicellulose and other ester-containing surface components, including wax-related species. The progressive changes in this peak with increasing temperature may indicate the deacetylation of hemicellulose during thermal treatment.^25^ Concurrently, the absorption bands in the regions 1020–1030 cm^−1^ and 1075–1155 cm^−1^, corresponding to C–O–C and C–O–H stretching vibrations in cellulose, respectively, also undergo intensity and positional changes, suggesting disruption of the polysaccharide matrix.^26^

**Fig. 4:**
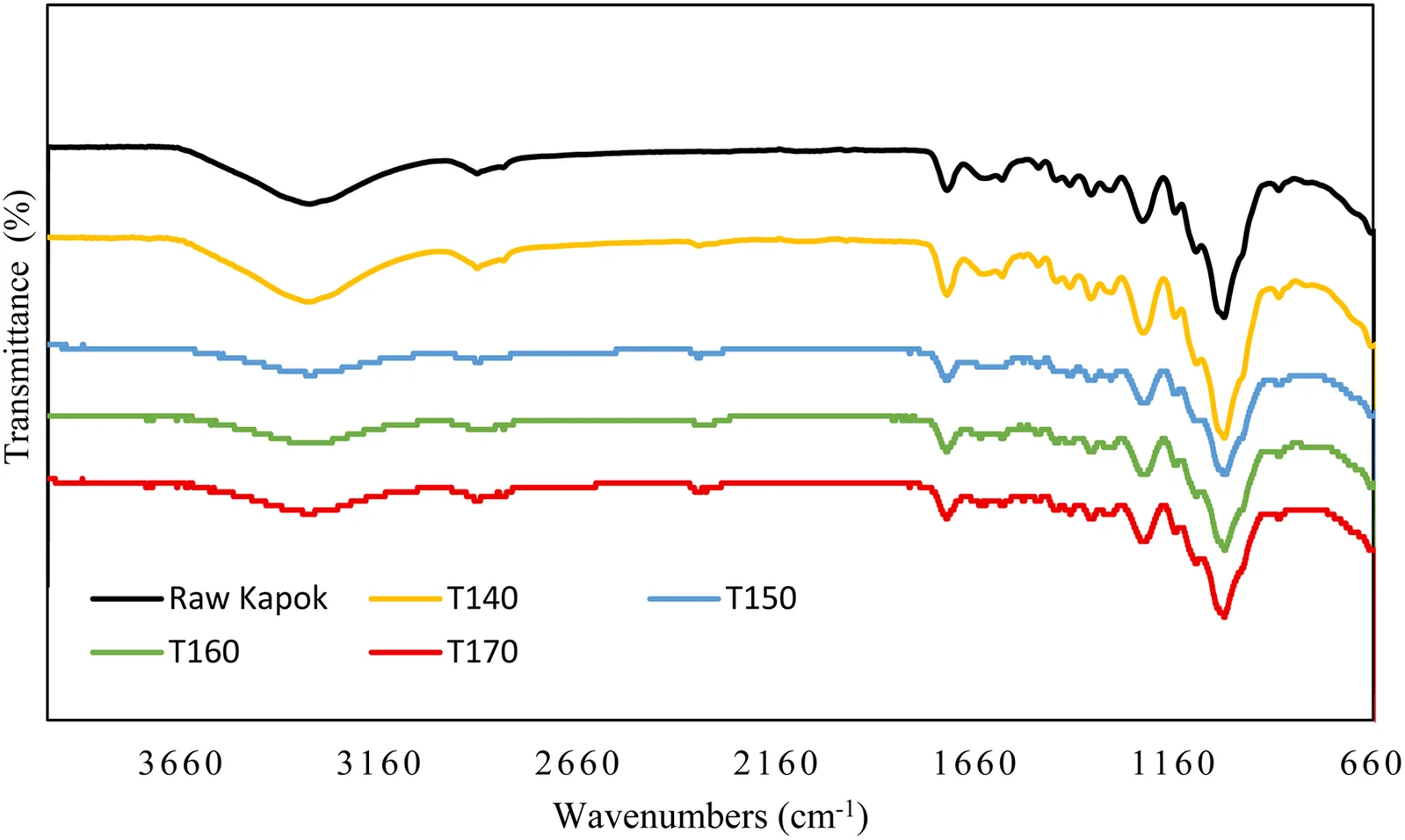
FTIR spectra of raw KF and nonwoven KF hot-pressed at different temperatures.

Additionally, the band around 1646 cm^−1^ is linked to adsorbed water in the fibers, while bands at 2886–2920 cm^−1^ are associated with C–H stretching in aliphatic structures, confirming the presence of cellulose and lignin components. The O–H bending near 1400–1450 cm^−1^ and the diminishing intensity of the 898 cm^−1^ peak related to β-glycosidic linkages in cellulose highlight the structural degradation of cellulose, likely catalysed by acidic byproducts of hemicellulose decomposition.^22,27^

At high temperatures, the FTIR spectra display more pronounced reductions in hydroxyl and carbonyl group absorptions, suggesting extensive chemical modification. The near disappearance of the 1730 cm^−1^ peak and greater suppression of the 898 cm^−1^ band indicate significant cellulose depolymerization and hemicellulose degradation. These findings are consistent with known mechanisms of thermal degradation in lignocellulosic materials, wherein heat induces cleavage of ester linkages and generation of volatile acidic compounds that further accelerate fiber matrix breakdown.

### 3.3. Grammage (GSM), thickness and density properties of nonwoven mat

Fig. 5 shows the influence of hydraulic hot-pressing temperature on the GSM of nonwoven KF mat. It can be seen from figure, that the GSM of the nonwoven KF mat decreases with increasing hot-pressing temperature, from 30.2 g m^−2^ at 140 °C to 27.8 g m^−2^ at 170 °C (≈7.95% reduction). This trend indicates that elevated temperatures promote fiber softening and partial thermal degradation, particularly in the amorphous cellulose regions, leading to increased fiber compaction. Similar effects of thermal processing on natural fibers have been reported in the literature.^28^

**Fig. 5:**
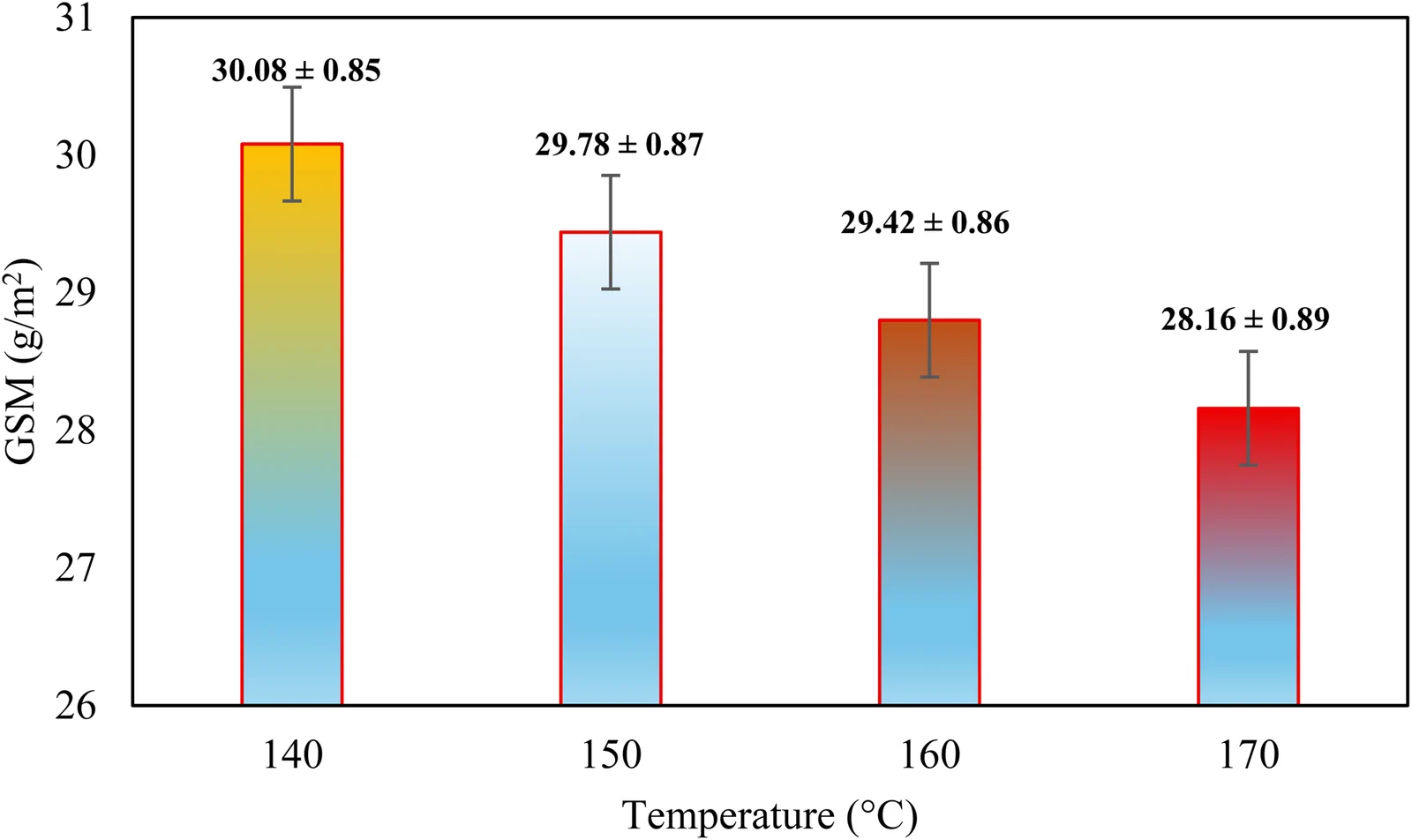
Influence of hydraulic hot-pressing temperature on the GSM of nonwoven KF mat.

Despite the reduction in GSM, the tensile strength increased with increasing hot-pressing temperature, as discussed in Section 3.4. This improvement is attributed to greater fiber consolidation at higher temperatures, resulting in closer fiber contact, reduced void content, and improved load transfer, as inferred from the observed morphology. Previous studies also confirm that processing conditions significantly influence the mechanical performance of nonwoven materials.^5^ GSM remains a critical factor in determining mechanical properties. Higher GSM generally improves tensile strength due to increased fiber content and better load distribution.^29–31^ However, in this case, improved consolidation at elevated temperatures compensates for the slight reduction in fiber mass. Excessive thermal exposure, however, may degrade cellulose and hemicellulose, potentially affecting long-term durability, even if short-term tensile strength appears improved. Therefore, careful control of processing conditions is essential.^21,32^ Additionally, increasing KF content has been shown to enhance mechanical strength.^27^ The Taguchi method was employed to optimize processing parameters, identifying 170 °C as the optimal temperature. This condition provides a balance between improved fiber compaction and minimal thermal degradation, supported by both experimental results and statistical analysis. Similar optimization approaches have been successfully applied in natural fiber composite studies.^33,34^ As presented in Fig. 5, the GSM of the KF nonwoven mats decreased progressively with increasing hydraulic hot-pressing temperature.

The effect of hot-pressing temperature on the thickness of the nonwoven KF mat is presented in Fig. 6. The thickness of the nonwoven mat gradually decreases with increasing hot-pressing temperature. The thickness decreased from 0.326 ± 0.031 mm at 140 °C to 0.250 ± 0.029 mm at 170 °C. This reduction can be attributed to the increased fiber rearrangement and compaction under heat and pressure, which reduced the void spaces within the nonwoven mat. The higher temperature enhanced fiber mobility and compressibility, resulting in a denser and thinner structure.^8,10^ As the temperature increases, the fibers become more pliable, allowing them to deform and pack more closely, thereby reducing the void spaces within the mat. In addition, the applied pressure promotes greater fiber-to-fiber contact and structural consolidation, resulting in a more compact and denser nonwoven structure with reduced thickness. Consequently, higher hot-pressing temperatures produce thinner mats due to improved fiber packing and densification.

**Fig. 6:**
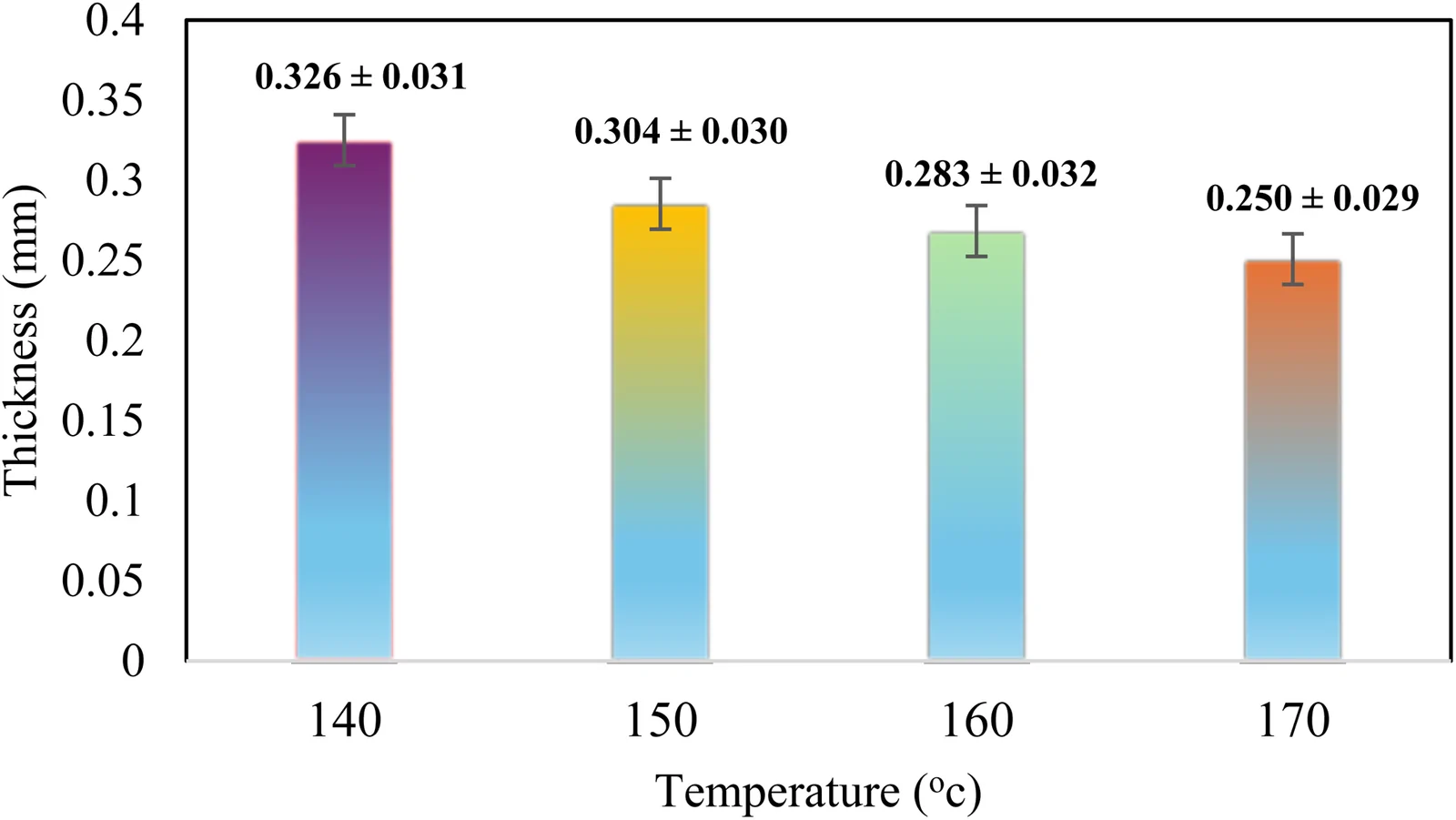
Thickness of nonwoven KF mats at various hot-pressing temperatures.

Fig. 7 illustrates the relationship between thickness and density. Between 140 and 170 °C, the mean thickness decreased from 0.326 to 0.250 mm, a reduction of approximately 23.3%, whereas the mean density increased from 0.370 to 0.450 g cm^−3^, an increase of approximately 21.6%. The inverse trend is consistent with progressive compaction of the fiber network.

**Fig. 7:**
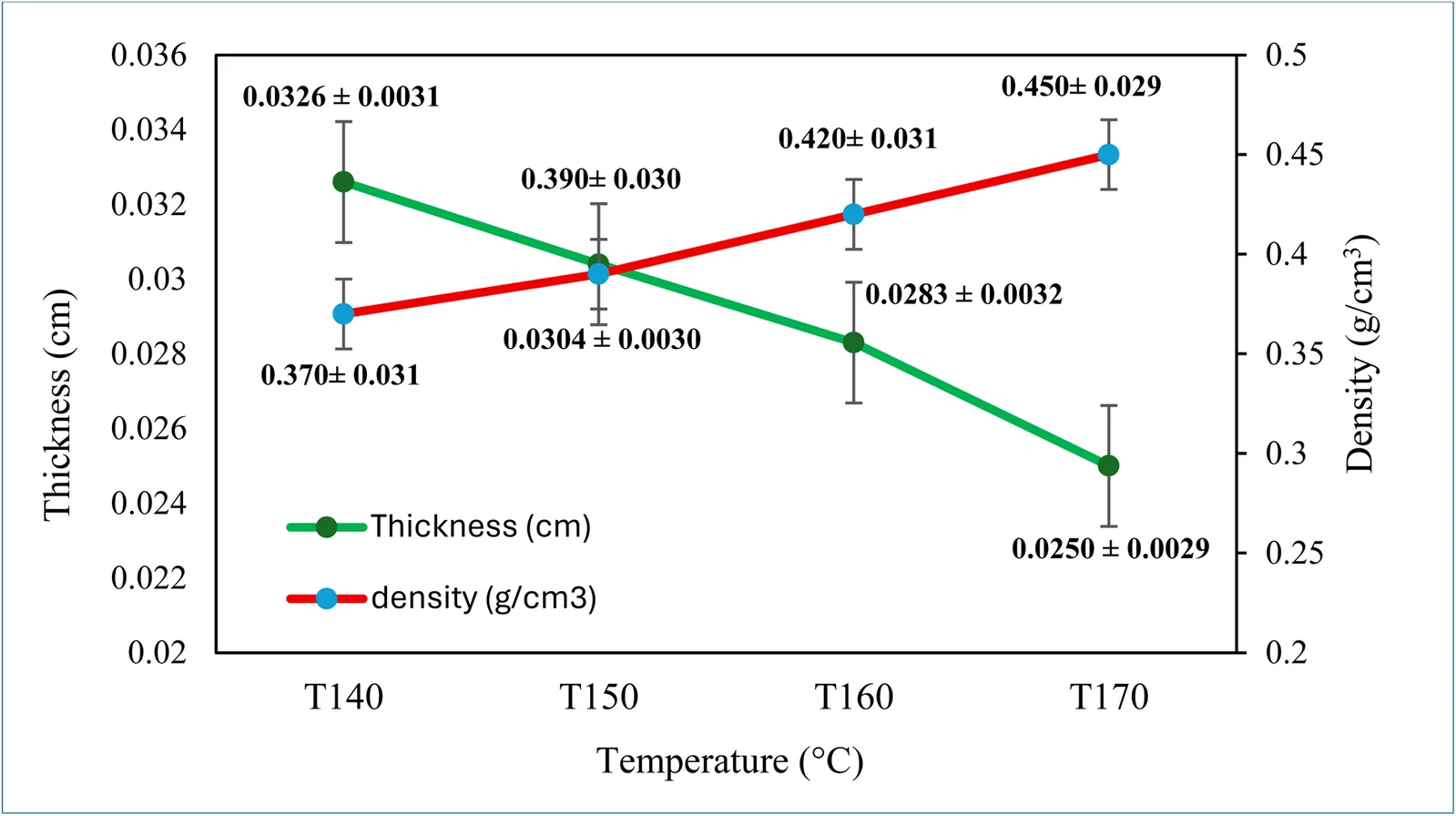
Relationship between thickness and density of nonwoven KF mats at various temperatures.

The observed decrease in thickness and corresponding increase in density with rising temperature suggests that hot-pressing induces significant fiber compaction. At 170 °C temperature, the thermal softening of amorphous components within kapok fibers such as hemicellulose facilitates structural rearrangement and collapse of inter-fiber voids. As a result, the fiber network becomes more tightly packed, leading to increased density and reduced mat thickness. These findings are consistent with earlier studies on thermally treated natural fiber materials.^35–37^

In morphological changes, the physical transformation of the mats has direct implications for functional performance. Higher density in nonwoven structures is often associated with enhanced mechanical properties, dimensional stability, and moisture resistance key attributes for industrial applications, particularly in marine environments.^38,39^ Thilagavathi *et al.* (2020) and Wang *et al.* (2023) reported that kapok-based composites with higher density exhibited better structural durability and water management properties, making them suitable for technical textile applications.^19,24^

The results in Table 2 show significant differences in GSM, thickness and density across the four temperature conditions (*p* < 0.001). Thus, the heat treatment significantly impacts on the physical properties of the KF mats.^35,37,41^ Values are reported as mean ± standard deviation for ten final specimens (*n* = 10) per condition. For the 25 cm × 25 cm final specimen area, the corresponding mean specimen masses were 1.88, 1.86, 1.84 and 1.76 g at 140, 150, 160 and 170 °C, respectively. The initial 8 g value described in Section 2.2 refers only to the gross as-carded web before hot pressing.

**Table 2:** Descriptive statistics of the physical properties of nonwoven mats prepared at different hot-pressing temperatures (*n* = 10)^a^

Temperature (°C)	Replicant number (*n*)	Weight (GSM) ± SD	Thickness (mm) ± SD	Density (g cm^−3^) ± SD
140	10	30.08 ± 0.85	0.326 ± 0.031	0.370 ± 0.031
150	10	29.78 ± 0.87	0.304 ± 0.030	0.390 ± 0.030
160	10	29.42 ± 0.86	0.283 ± 0.032	0.420 ± 0.031
170	10	28.16 ± 0.89	0.250 ± 0.029	0.450 ± 0.029

a Note: values are expressed as mean ± standard deviation (SD). *n* = 10 specimens were tested for each hot-pressing temperature.

Table 3 displays the ANOVA results for GSM, thickness (mm), and density (g cm^−3^) of nonwoven KF mats subjected to heat treatment at temperatures of 140 °C, 150 °C, 160 °C, and 170 °C. The table includes the sum of squares, degrees of freedom (df), mean square, *F*-value, and *p*-value for between-group and within-group variations. The *p*-values are below 0.001 for all three properties, indicating statistically significant differences among the temperature groups. Tukey's HSD comparisons are summarised in Table 6.

**Table 3:** One-way ANOVA results for the physical properties of nonwoven mats at different hot-pressing temperatures

Properties	Source	df	Sum of squares	Mean square	*F*-value	*p*-value
Weight (GSM)	Between groups	3	12.47	4.16	12.34	<0.001
	Within groups	36	13.53	0.38		
	Total	39	26			
Thickness (mm)	Between groups	3	0.015	0.005	20.56	<0.001
	Within groups	36	0.009	0.00025		
	Total	39	0.024			
Density (g cm^−3^)	Between groups	3	0.035	0.0117	15.89	<0.001
	Within groups	36	0.026	0.00072		
	Total	39	0.061			

### 3.4. Mechanical properties of nonwoven mats

Fig. 8 and 9 showed the tensile strength and tensile modulus of nonwoven KF mat at four different temperatures (140 °C, 150 °C, 160 °C, and 170 °C). A progressive and substantial increase in both mechanical parameters was observed as hot-pressing temperature increases.

**Fig. 8:**
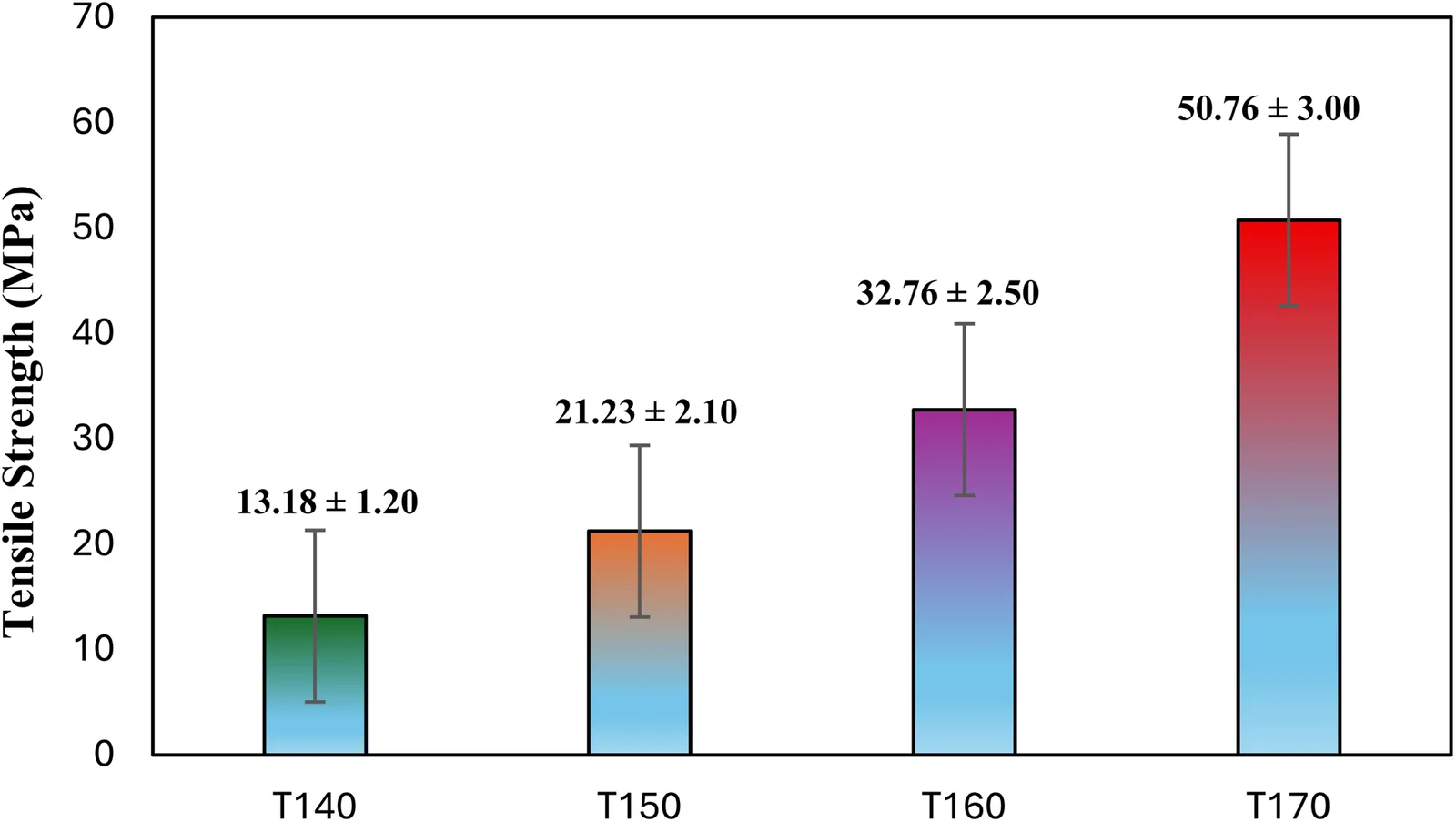
Tensile strength of nonwoven KF mats produced at different hot-pressing temperatures.

**Fig. 9:**
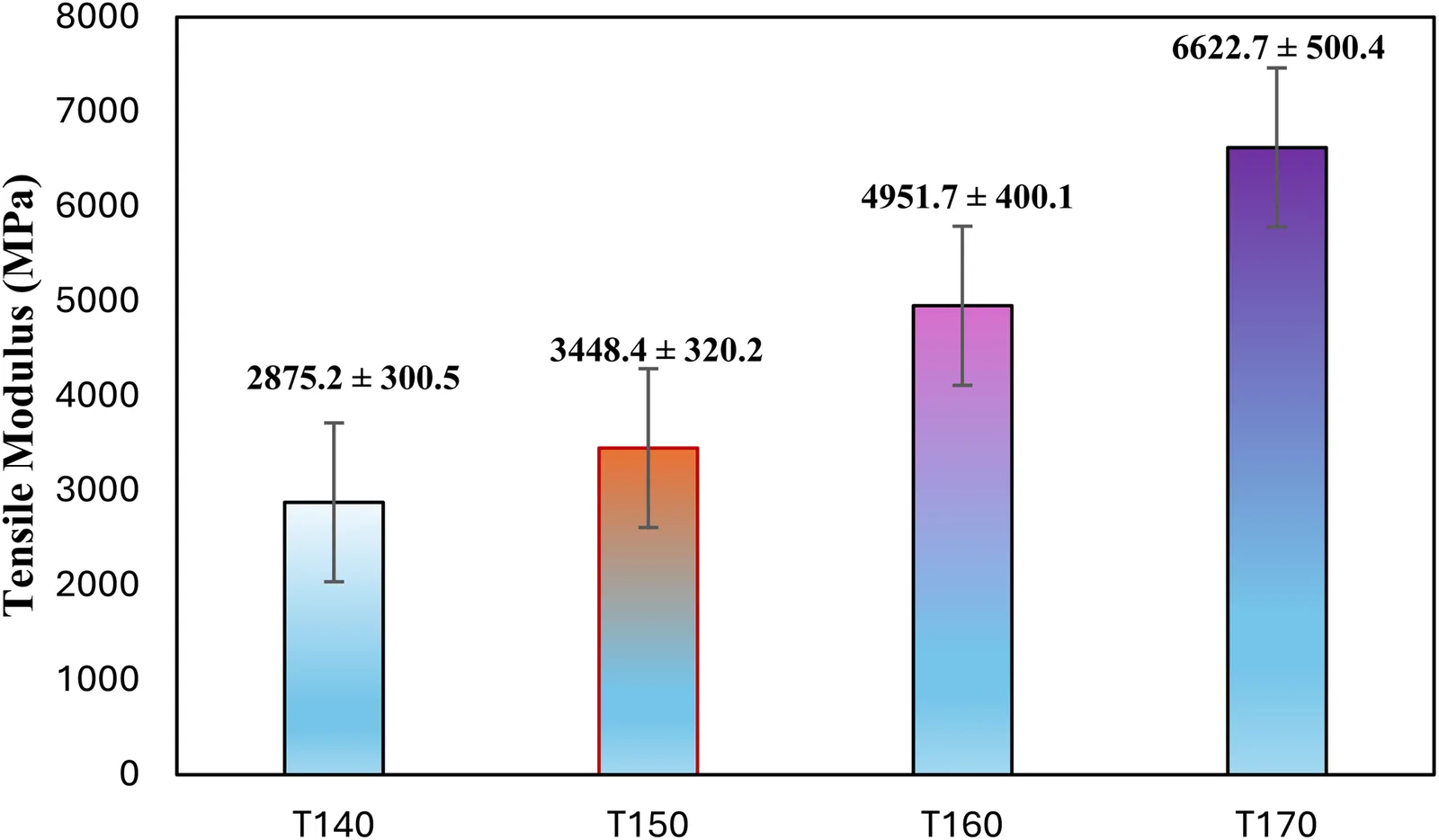
Tensile modulus of nonwoven KF mats produced at different hot-pressing temperatures.

Based on result in Fig. 8 and 9, at 140 °C, the tensile strength and modulus were recorded at approximately 13 MPa and 2875 MPa, respectively. This relatively low mechanical performance indicates inadequate inter-fiber interlink, which can be attributed to insufficient thermal softening at this temperature. The applied pressure alone is not enough to promote effective inter-fiber contact or reduce void content significantly. This finding is in agreement with the study by Pickering and Le (2016), which reported that low-temperature consolidation often leads to weak KF consolidation in natural fiber composites due to limited molecular mobility and poor fiber-matrix adhesion.^42^

At 150 °C, tensile strength increased to 21 MPa, and tensile modulus rose to 3448 MPa. This improvement can be associated with enhanced fiber softening, promoting better compaction and increased consolidation between kapok fibers. Mohammed *et al.* (2015) reported similar behaviour in natural fiber laminates, where moderate thermal input improved consolidation and decreased porosity.^25^ However, although improvement is notable, the microstructure at this temperature still exhibits some unbonded regions and minor voids, indicating that consolidation is not yet fully optimized.

At 160 °C, the tensile strength of KF mat is reaching 32.8 MPa and modulus improving to 4952 MPa. This phase signifies a transition point where thermal energy is sufficient to soften the fiber surfaces effectively, allowing better molecular entanglement and stress transfer. Dejene, 2024 demonstrated that fiber crystallinity and stiffness improve under controlled thermal exposure, contributing to mechanical gains in bio-composites.^43^ The reduced void ratio and more uniform stress distribution across the mat are likely contributing factors to the significant rise in both strength and modulus.

The best mechanical performance was achieved at 170 °C, where tensile strength and modulus peaked at 50.76 MPa and 6622.7 MPa, respectively. This reflects a 292.31% increase in strength relative to the 140 °C baseline. The consistent upward trend confirms that elevated pressing temperatures promote superior consolidation, densification, and interfacial consolidation. Microscopic examination of the surface micrographs at this condition revealed a more compact, homogenous matrix with minimal voids an indication of well-developed inter-fiber adhesion. Watanabe *et al.* (1999) observed similar result for natural fiber composites.^44^ However, it is important to note that further increases in temperature beyond 170 °C are likely to result in cellulose degradation, as preliminary trials indicated discolouration and thermal damage to the fiber structure. It has warned of such deterioration, which can offset the mechanical gains achieved through processing.^13^

The critical role of temperature control in regulating fiber consolidation, resin flow, and mechanical integrity in natural fiber systems as observed by other reseachers.^45–47^ The clear correlation between temperature and tensile performance underscores the need for careful process control to maximise mechanical output while avoiding structural compromise. Finally, 170 °C is identified as the optimal hot-pressing temperature for nonwoven KF mats.

The mechanical properties of nonwoven KF at varying temperatures with each data set averaged over ten replicates for statistical reliability are presented in Table 4. While previous sections addressed the marked increases in tensile strength and modulus, the break percentage strain (% strain at fracture) also exhibited a consistent upward trend with increasing processing temperature. Specifically, the break strain increased from 0.63% at 140 °C to 0.95% at 170 °C representing an approximate enhancement of 51.5%. This trend indicates a modest but meaningful improvement in the fiber interlock capacity to elongate before rupture, suggesting that elevated temperatures enhance ductility alongside strength and stiffness. The incremental increases observed 0.88% at 150 °C, 0.89% at 160 °C, and culminating at 0.95% at 170 °C imply a thermally induced modification in fiber entanglement and matrix cohesion, possibly due to enhanced inter-fiber consolidation and partial lignin softening, as noted in comparable studies on thermally processed natural fiber composites.^48^

**Table 4:** Descriptive statistics of tensile properties of nonwoven mats prepared at different hot-pressing temperatures (*n* = 10)

Temperature (°C)	*n*	Tensile strength (MPa) mean ± SD	Tensile modulus (MPa) mean ± SD	Elongation at break (%) mean ± SD
140	10	13.18 ± 1.20	2875.2 ± 300.5	0.630 ± 0.05
150	10	21.23 ± 2.10	3448.4 ± 320.2	0.880 ± 0.06
160	10	32.76 ± 2.50	4951.7 ± 400.1	0.900 ± 0.05
170	10	50.76 ± 3.00	6622.7 ± 500.4	0.954 ± 0.04
Overall	40	29.48 ± 15.55	4336.8 ± 1601.7	0.841 ± 0.131

These results suggest that thermal treatment not only improves the mechanical resistance of KF but also promotes a slight increase in flexibility and energy absorption prior to failure. This modest increase in strain at break is particularly relevant for applications where both strength and toughness are critical. The one-way ANOVA confirmed that the observed differences in break strain across temperature groups were statistically significant (*p* < 0.05), further validating the influence of processing conditions on mechanical performance. Overall, the improvement in break strain, together with the previously observed enhancements in strength and stiffness, highlights the importance of hot-pressing temperature in optimizing the mechanical behaviour of KF nonwovens, consistent with findings reported by Srisuk *et al.* (2024) and Wang *et al.* (2023) in recent studies on bio-based material processing.^19,49^

The slight increase of elongation at break should be considered in the light of the results of tensile strength and modulus. The one-way ANOVA showed that temperature had a significant effect on elongation at break (*p* < 0.005 in Table 5), and Tukey's HSD showed that the most apparent pairwise differences were those involving the 140 °C group. No specific test for toughness or energy absorption was performed.

**Table 5:** One-way ANOVA results for the tensile properties of nonwoven mats at different hot-pressing temperatures

Property	Source of variation	df	Sum of squares	Mean square	*F*-Value	*p*-Value
Tensile strength (MPa)	Between groups	3	2451.13	817.04	18.76	<0.001
	Within groups	36	1565.60	43.49	—	—
	Total	39	4016.73	—	—	—
Tensile modulus (MPa)	Between groups	3	36 842 073	12 280 691	22.45	<0.001
	Within groups	36	19 702 243	547 284.5	—	—
	Total	39	56 544 316	—	—	—
Elongation at break (%)	Between groups	3	0.2300	0.0767	5.63	<0.005
	Within groups	36	0.4902	0.0136	—	—
	Total	39	0.7202	—	—	—

The elongation at break improvement is small and should not be used as a measure of improvement in toughness or energy absorption. The result is given as a change in elongation at break only and the interpretation is limited to the geometry of the tested specimen and short-term tensile response.

Table 5 presents the results of a one-way ANOVA examining the influence of hot-pressing temperature (ranging from 140 °C to 170 °C) on the mechanical properties of nonwoven kapok fiber mats. Each temperature condition was replicated with ten samples to ensure statistical reliability and meaningful interpretation of the variance within and between groups.

The analysis reveals that pressing temperature has a statistically significant effect on all three mechanical parameters evaluated: tensile strength, tensile modulus, and break percentage strain. Specifically, the tensile strength increased from 13.18 MPa at 140 °C to 50.76 MPa at 170 °C, with an *F*-ratio of 37.18 and *p*-value < 0.001, indicating a highly significant difference between group means. This substantial enhancement reflects a progressive strengthening of inter-fiber bonds as thermal energy facilitates more effective molecular mobility and fiber rearrangement.

The tensile modulus exhibited a marked increase, rising from 2875.2 MPa to 6622.7 MPa, with an *F*-ratio of 19.46 (*p* < 0.001). This trend demonstrates that the stiffness of the KF mat structure improves significantly under elevated temperatures. The enhancement is likely attributed to reduced porosity and increased packing density, which constrain fiber mobility and allow for improved stress transfer across the network. Besides that, the break percentage strain also showed a statistically significant rise from 0.63% to 0.95% with an *F*-ratio of 5.63 and *p*-value < 0.005. This moderate improvement suggests that higher temperatures not only increase strength and stiffness but also slightly enhance the ductility of the material. A better-distributed internal stress profile across a more cohesive fiber structure may account for this behaviour.

These quantitative outcomes are confirmed by qualitative observations from optical micrographs taken at each temperature level. At lower pressing temperatures (140 °C and 150 °C), the fiber network appears loosely arranged with visible interstitial gaps and limited inter-fiber fusion. In contrast, at 160 °C and more distinctly at 170 °C, the fiber structure appears more consolidated, with minimal voids and improved fiber contact, suggesting enhanced thermal consolidation. This visual confirmation reinforces the interpretation that mechanical improvements result from microstructural transformation induced by controlled thermal exposure. The relation between statistical significance and morphology validates the conclusion that hot-pressing temperature is a dominant factor influencing the mechanical integrity of kapok-based nonwovens. Thermal treatment facilitates improved fiber–matrix interaction, consolidation, and load distribution in natural fiber composites as observed by Wang *et al.* (2023).^19^

By integrating statistical evidence and microstructural validation, this study demonstrates that 170 °C is the optimal hot-pressing temperature to maximise the tensile strength, modulus, and ductility of nonwoven kapok fibers without inducing thermal degradation. This balance between mechanical enhancement and material integrity forms the foundation for the subsequent development of kapok-based hybrid composites for high-performance applications.

Hot-pressing temperature had a statistically significant effect on tensile strength, tensile modulus and elongation at break. Tensile strength increased from 13.18 MPa at 140 °C to 50.76 MPa at 170 °C (*F* = 18.76, *p* < 0.001). Tensile modulus increased from 2875.2 to 6622.7 MPa (*F* = 22.45, *p* < 0.001). Elongation at break increased from 0.630% to 0.954% (*F* = 5.63, *p* < 0.005). These results demonstrate a statistically significant temperature effect, while the mechanism is discussed cautiously in terms of compaction, fiber contact and load transfer inferred from the morphology.

The Tukey HSD results in Table 6 identify the temperature pairs responsible for the significant omnibus effects. Tensile strength differed significantly between every temperature pair. Tensile modulus did not differ significantly between 140 and 150 °C but differed for the remaining pairs. For elongation at break, the significant comparisons involved 140 °C *versus* each higher temperature, whereas the comparisons among 150, 160 and 170 °C were not significant at *α* = 0.05.

**Table 6:** Summary of Tukey's HSD pairwise comparisons at *α* = 0.05

Property	Significant pairs (*p* < 0.05)	Not significant (*p* ≥ 0.05)
GSM	140–170; 150–170; 160–170	140–150; 140–160; 150–160
Thickness	140–150; 140–160; 140–170; 150–160; 150–170; 160–170	None
Density	140–160; 140–170; 150–170	140–150; 150–160; 160–170
Tensile strength	140–150; 140–160; 140–170; 150–160; 150–170; 160–170	None
Tensile modulus	140–160; 140–170; 150–160; 150–170; 160–170	140–150
Elongation at break	140–150; 140–160; 140–170	150–160; 150–170; 160–170

Together, the statistical and morphological observations support a temperature-dependent change in the consolidation state of the KF network. They do not establish polymerisation, a continuous fiber matrix or direct chemical bonding. The results are limited to the tested specimen geometry and short-term measurements. Within the investigated range, 170 °C produced the highest measured tensile strength and modulus. It is therefore identified as the best-performing tested condition, not as a global optimum or universally safe temperature. Further temperatures and thermal/durability analyses are needed to define the processing window for a specific application.

Table 7 represents the physical and mechanical properties of kapok-based nonwovens and related kapok fiber-reinforced composites reported in the literature.

**Table 7:** Comparison of physical and mechanical properties of kapok-based nonwovens and related composites^a^

Nonwoven/material	Composition	Fabrication method	GSM (g m^−2^)	Thickness (mm)	Tensile strength (MPa)	References
Kapok nonwoven mat	100% kapok fiber	Hot-pressing at 140–160 °C	28.16–30.08	0.250–0.326	29.48	This study
Carded kapok nonwoven	100% kapok fiber	Hot-pressing; 160–180 °C, Taguchi design	NR	NR	22.12–37.69	10
Kapok/waste-silk nonwoven	0% kapok + 100% waste silk	Wet-laid + hot pressing	34	0.287	0.58	19
Kapok/waste-silk nonwoven	25% kapok + 75% waste silk	Wet-laid + hot pressing	34	0.242	NR	19
Kapok/waste-silk nonwoven	50% kapok + 50% waste silk	Wet-laid + hot pressing	34	0.214	NR	19
Kapok/waste-silk nonwoven	75% kapok + 25% waste silk	Wet-laid + hot pressing	34	0.157	NR	19
Kapok/waste-silk nonwoven	100% kapok + 0% waste silk	Wet-laid + hot pressing	34	0.094	10.85	19
Kapok/PP needle-punched nonwoven	Kapok/PP = 70/30, 60/40 or 50/50	Carding + needle punching	200–400	1.3–3.6	NR	40
Raw kapok/epoxy composite	5 wt% kapok fiber + epoxy	Hand lay-up	NR	NR	17.62	50
Dewaxed kapok/epoxy composite	5 wt% dewaxed kapok fiber + epoxy	Dewaxing + hand lay-up	NR	NR	23.19	50
Dewaxed kapok/epoxy composite	Kapok fiber + epoxy	Dewaxing treatment	NR	NR	NR	51
Silane-treated kapok/epoxy composite	Kapok fiber + epoxy	Dewaxing + silane treatment + hand lay-up	NR	NR	NR	52
Kapok/epoxy composite	1–7 wt% kapok fiber + epoxy	Hand lay-up	NR	NR	∼11–18	53
Kapok/bran/epoxy composite	150 g kapok + 25 g bran + 150 g epoxy	Hand lay-up	NR	NR	34.02	54
Kenaf/kapok/polyester hybrid composite	Kenaf + kapok + polyester	Hand lay-up	NR	NR	25.25	55

a Note: NR = not reported in the cited publication.

A comparison shows that only few studies have been reported on pure KF nonwoven mats, and mostly KF composites with polymer matrices were studied. The physical and mechanical properties of the pure or hybrid kapok nonwoven mat are generally lower than polymers-matrix composites due to the absence of a continuous matrix to provide effective stress transfer and interfacial bonding. The mechanical performance of the binder-free nonwoven structure is mainly governed by the fiber entanglement, friction, packing density and degree of consolidation achieved during hot pressing. Although having lower mechanical properties, the pure KF nonwoven mats have advantages of low density, biodegradability and no additional polymeric binder. These properties make them potentially suitable for lightweight and sustainable applications. Therefore, the limited literature on pure KF nonwoven mats suggests the need for additional research and optimization of the processing conditions to improve their structural and mechanical properties.^10,19,50–55^

### 3.5. Limitations

This study has several limitations. The investigation was restricted to a hot-pressing temperature range of 140–170 °C, and a no-PET control was not included; therefore, the independent contribution of the temporary PET release films cannot be completely separated from the hot-pressing effect. The exact heating and cooling rates and the pressure and foot area used for thickness measurements should also be verified from the original experimental records. In addition, the FTIR spectra were not documented as baseline-corrected and normalized, limiting quantitative comparison of spectral intensities. The study focused on specimen-level physical, structural, thermal, and mechanical properties; long-term durability, environmental ageing, fatigue behaviour, moisture resistance, direct inter-fiber bonding, and fracture-surface characteristics were not investigated. Finally, the laboratory-scale carding and hot-pressing process was not validated at panel or industrial scale. Therefore, the findings should be interpreted within the tested conditions, and further application-specific, panel-scale, and full-scale testing is required before confirming suitability for specific engineering applications.

## 4. Conclusions

It can be concluded that KF nonwoven mats were successfully produced in an Ashford drum carder and then hydraulic hot pressed. The hot-pressing temperature was found to be an important factor for the physical, morphological, structural and mechanical properties of the nonwoven mats fabricated. The GSM decreased gradually from 30.2 to 27.8 g m^−2^ (about 7.95%) with the increase of hot-pressing temperature from 140 to 170 °C, and the mat thickness decreased, which resulted in the increase of the density by means of better fiber consolidation. The surface morphology was observed to be increasingly smooth and compact fiber structures with increasing temperature. FTIR analysis revealed a decrease in the intensity of the –OH absorption peaks, indicating partial thermal modification of the fiber surface. The hot-pressing temperature had significant influence on mechanical properties of the mat. In particular, the nonwoven mat at 170 °C showed a significant improvement in performance with a tensile strength of 50.76 MPa and a tensile modulus of 6622 MPa. However, preliminary tests showed discoloration and thermal damage to the fiber structure, so higher temperatures above 170 °C are unlikely to be suitable. Statistical analysis confirmed the effect of hot-pressing temperature on the physical and mechanical properties of nonwoven mats was statistically significant. The results overall suggested that hot pressing can be regarded as an effective and environmentally friendly post-processing technique to enhance the structural integrity and mechanical performance of KF nonwoven mats. The developed KF nonwoven mats show potential for selected lightweight and sustainable applications, but further application-specific mechanical, durability and long-term performance evaluations are needed to establish their suitability for specific engineering or industrial uses.

## Author contributions

M. A. M. Mohd Idrus: conceptualization, data curation, writing – original draft. Md. Saiful Islam: conceptualization, supervision, revise Mohd Yuzri Mohd Yusop: review & editing Zulzamri Salleh: formal analysis, and review.

## Conflicts of interest

There are no conflicts to declare.

## Data Availability

The data that support the findings of this study are available from the corresponding author upon reasonable request.
